# Supplementary figures and images for: Bimodal expression of Type 3 Secretion System 2 enables cooperative virulence among intracellular Salmonella Typhimurium
Source: PLoS Pathog. 2025 Dec 1;21(12):e1013728. doi: 10.1371/journal.ppat.1013728 (PMC12677785; doi:10.1371/journal.ppat.1013728)

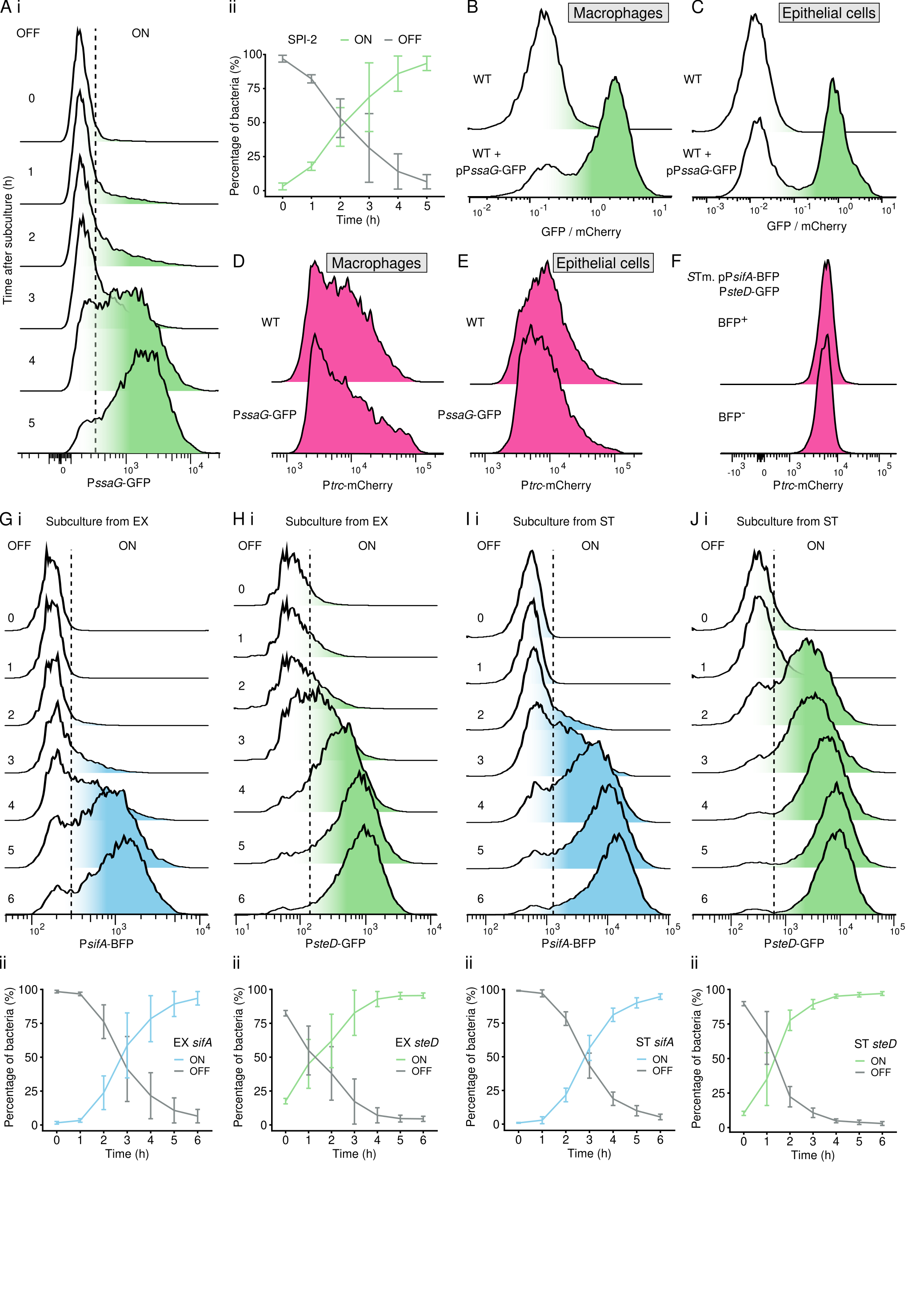

Supplement: S1 Fig — (A) PssaG activity over time. (i) GFP expression in STm WT + pPssaG-GFP was analysed by flow cytometry at indicated time after subculture from late exponential phase (LB, OD 1.8) into MgMES pH 5. (ii) Quantification of GFP+ (ON) and GFP- (OFF) bacteria as represented in (i). Data are from 3 independent experiments in technical triplicates and show means ± SD. (B and C) Bimodality of PssaG activity in macrophages (B) and epithelial cells (C) is not due to general expressional difference. Data from Figure 1H and I were reanalysed to show GFP/mCherry MFI ratio. Constitutively expressed mCherry from Ptrc was used as a Salmonella marker and for normalization to overall transcriptional activity. STm WT not carrying the pPssaG-GFP were used as a negative control. (D and E) Presence of the reporter system does not represent major burden for intracellular bacteria in RAW264.7 macrophages (D) and Mel JuSo epithelial cells (E). Representative flow cytometry data from 3 independent experiments comparing bacterial amounts of STm WT + pPssaG-GFP and STm WT. (F) Bimodality of PsifA and PsteD activity in macrophages is not due to general expressional difference. Data from Fig 2E were separated to BFP+ (BFP+ with BFP+GFP+ populations) and BFP- (BFP-GFP- and GFP+ populations). Constitutively expressed mCherry from Ptrc was used as a Salmonella marker and for normalization to overall transcriptional activity. (G) PsifA activity over time. (i) BFP expression in STm WT + pPsifA-BFP_PsteD-GFP was analysed by flow cytometry at indicated time after subculture from late exponential phase (OD 1.8 in LB) into MgMES pH 5. (ii) Quantification of BFP+ (ON) and BFP- (OFF) bacteria as represented in (i). Data are from 3 independent experiments in technical triplicates and show means ± SD. (H) PsteD activity over time. (i) GFP expression in STm WT + pPsifA-BFP_PsteD-GFP was analysed by flow cytometry at indicated time after subculture from late exponential phase (OD 1.8 in LB) into MgMES pH 5. (ii) [file ppat.1013728.s001.tiff]

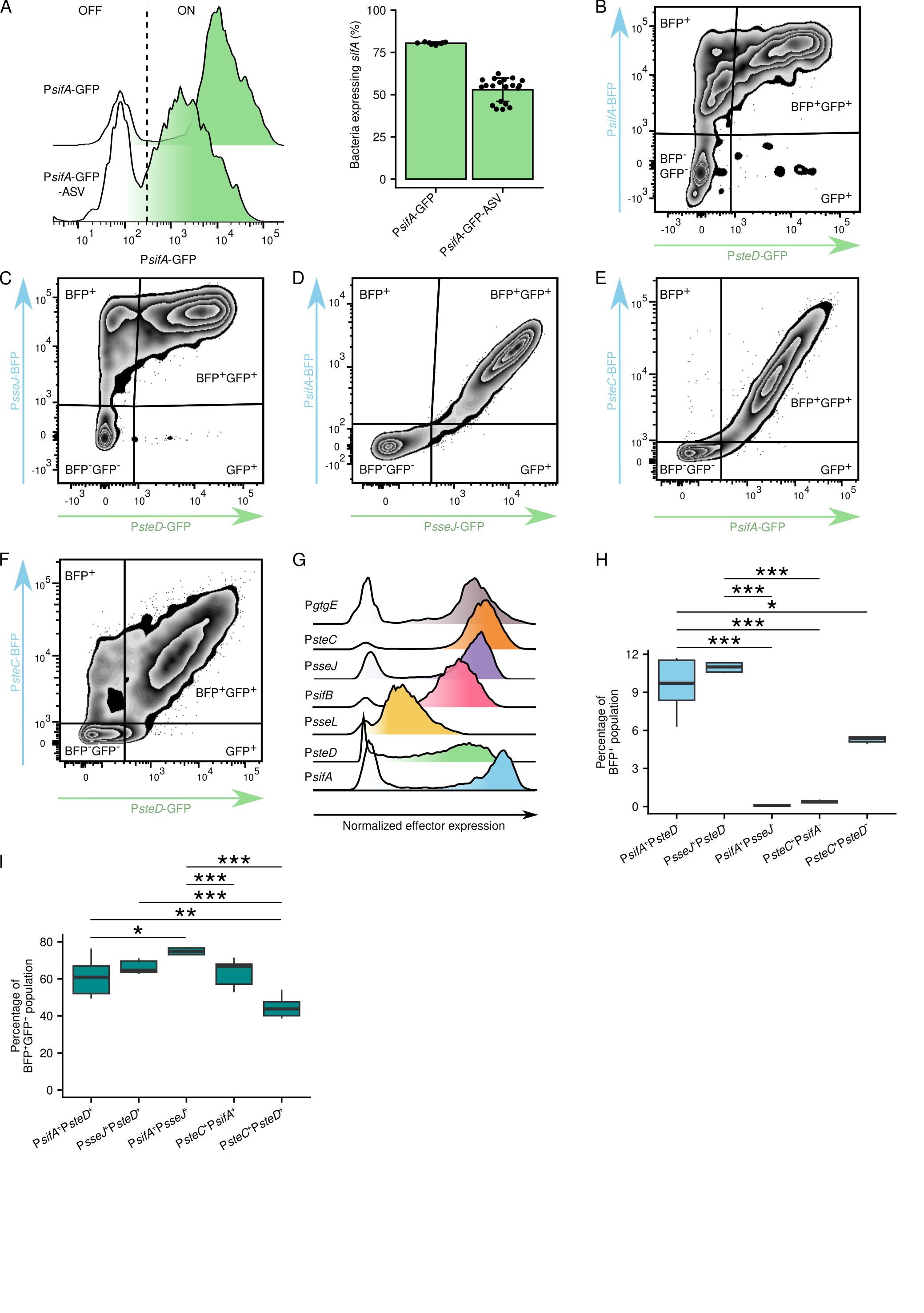

Supplement: S2 Fig — (A) Bimodality of PsifA activity measured using destabilized reporter in epithelial cells. Mel JuSo cells were infected with STm WT + pPsifA-GFP, or STm WT + pPsifA-GFP-ASV for 24 h before hypotonic lysis. PsifA activity was monitored as GFP fluorescence in bacteria from cell lysate by flow cytometry. The bar plot represents quantification of bacteria in depicted gates. Data are from at least 3 independent experiments in at least technical duplicates and show means ± SD. (B-I) Activity of PsifA, PsteD, PsseJ, PsteC, PgtgE and PsifB in epithelial cells. Mel JuSo cells were infected for 24 h before hypotonic lysis. Promoter activity was monitored as BFP and GFP fluorescence, respectively, in bacteria from cell lysate by flow cytometry. Representative zebra plots (B-F) of STm WT + pPsifA-BFP_PsteD-GFP (B), STm WT + pPsseJ-BFP_PsteD-GFP (C), STm WT + pPsifA-BFP_PsseJ-GFP (D), STm WT + pPsteC-BFP_PsifA-GFP (E) and STm WT + pPsteC-BFP_PsteD-GFP (F) after infection. (G) Bimodality of PgtgE, PsteC, PsseJ, PsifB, PsseL, PsteD, PsifA activity in epithelial cells. Mel JuSo cells were infected with STm WT + pPeffector-BFP_PsteD-GFP (where the native promoter for gtgE, steC, sseJ, sifB, sseL, or sifA controlled BFP expression respectively) for 24 h before hypotonic lysis. Promoter activity was monitored as BFP and GFP fluorescence, respectively, in bacteria from cell lysate by flow cytometry. The histograms show BFP fluorescence of individual promoters normalized to GFP fluorescence from the same reporter to allow direct comparison between individual reporters. (H, I) Summarising box and whiskers plot of percentages of BFP+ (G) and BFP+GFP+ (H) populations. Data from 2-4 independent experiments in technical mono- to triplicates and show medians, Q1 and Q3. *p < 0.05; **p < 0.01; ***p < 0.001 (One-way ANOVA with Tukey HSD test). (TIFF) [file ppat.1013728.s002.tiff]

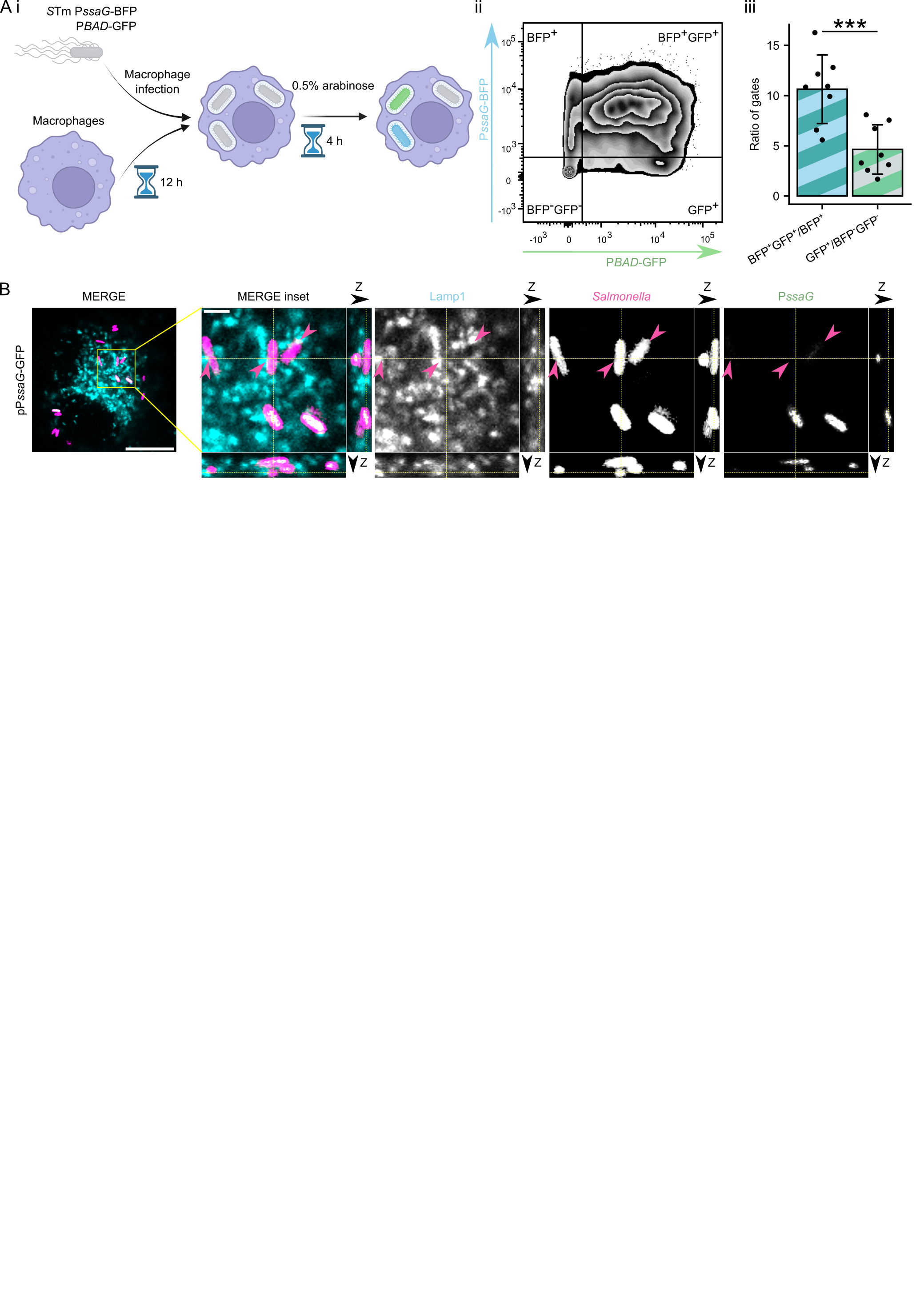

Supplement: S3 Fig — (A) SPI-2OFF Salmonella are metabolically active. (i) Schematic of assessment of metabolic activity of SPI-2OFF Salmonella. RAW264.7 macrophages were infected with STm WT + pPssaG-BFP_PBAD-GFP for 12 h. Subsequently, arabinose was added to the cell media to assess the capacity of intracellular bacteria to metabolize arabinose. PssaG and PBAD activity was measured as BFP and GFP fluorescence, respectively, by flow cytometry 4 h after arabinose addition. (ii) Representative flow cytometry data for PssaG and PBAD activity in individual bacteria residing in RAW264.7 macrophages treated as described in (i). (iii) The bar charts show ratios of percentages from populations BFP+GFP+ and BFP+, or GFP+ and BFP-GFP-. Data are from 3 independent experiments in technical triplicates and show means ± SD. ***p < 0.001 (Paired two-sample t-test). Created in BioRender. Pospíšilová, M. (2025) https://BioRender.com/ex2ztjy. (B) SPI-2OFF Salmonella is in SCV. Representative confocal fluorescence microscopy image of GFP expression in STm WT + pPssaG-GFP in Mel JuSo cells expressing LAMP1-mTurqoise 24 h p.i. The images show a single Z axis layer and X-Z and Y-Z projections (at the bottom and side of the main images, respectively) to show the signal of the SCV marker LAMP1 adjacent to SPI-2OFF Salmonella. The arrowheads indicate SPI-2OFF (GFP-) bacteria surrounded with LAMP1. Scale bar, 10 µm in the overview image and 2 µm in the magnified inset images. (TIFF) [file ppat.1013728.s003.tiff]

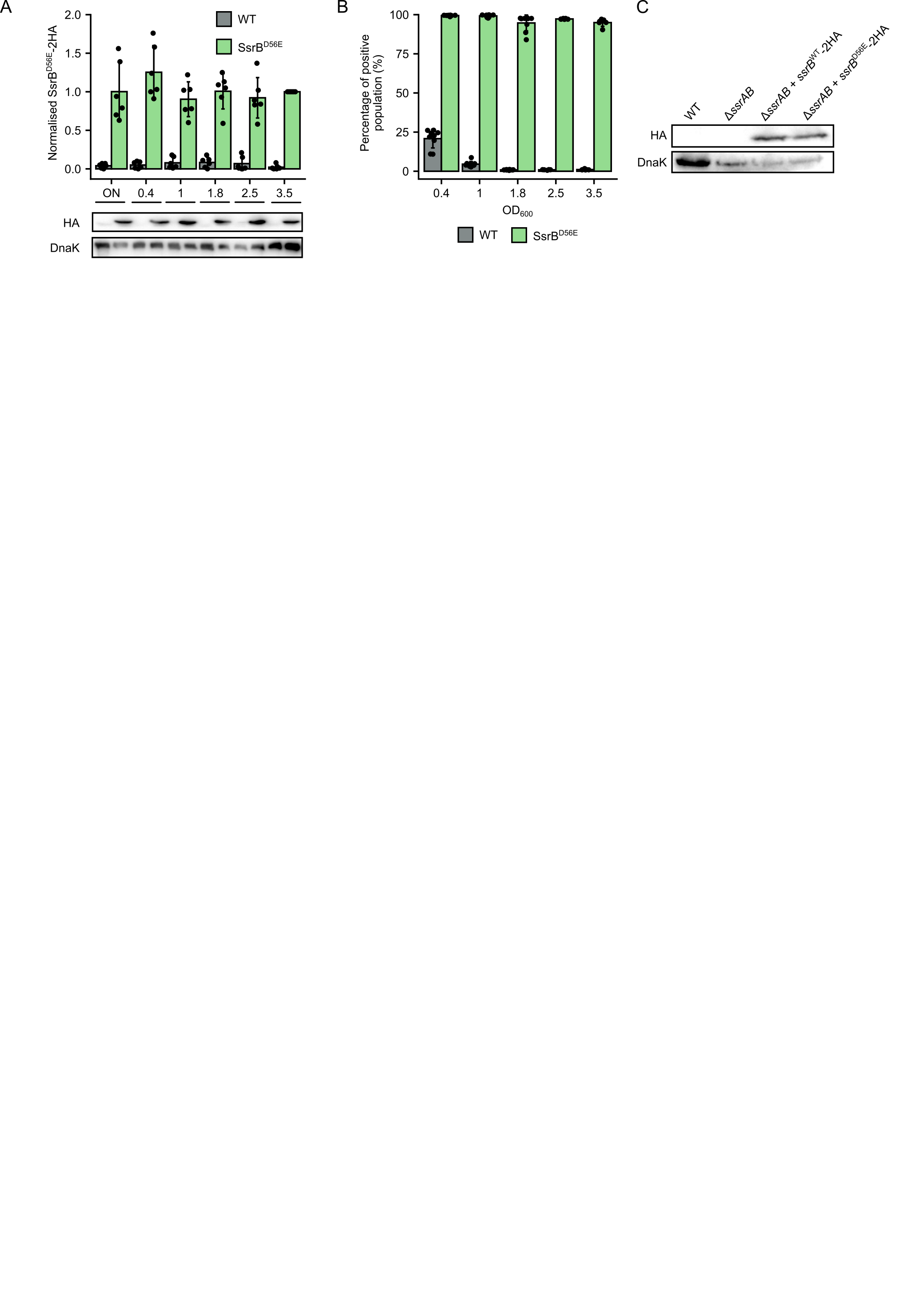

Supplement: S4 Fig — (A) The amount of SsrBD56E-2HA expressed from Ptrc is constant throughout the growth of Salmonella culture. Total levels of SsrBD56E-2HA in STm WT + pPtrc-ssrBD56E-2HA + pPssaG-GFP (SsrBD56E) cultures used in Fig 4A was examined for the presence of HA tag using immunoblotting at indicated OD600 after subculture from a late stationary phase (16 h in LB) into fresh LB medium and measured at defined OD. STm WT + pPssaG-GFP (WT) was used as a negative control and anti-DnaK (Salmonella) antibody was used for loading control. Representative of 3 independent experiments and quantification of HA signal relative to DnaK signal intensities from 3 experiments represented is shown. Data show means ± SD. (B) PssaG activity is constant in the presence of SsrBD56E-2HA. GFP expression in STm WT + pPssaG-GFP (WT) and STm WT + pPtrc-ssrBD56E-2HA + pPssaG-GFP (SsrBD56E) was analysed by flow cytometry in samples from A) at indicated OD600. Data are from 3 independent experiments in technical triplicates and show means ± SD. (C) The amount of SsrB-2HA and SsrBD56E-2HA is comparable during infection. RAW264.7 macrophages were infected with STm ΔssrAB + pPtrc-ssrB-2HA + pPssaG-GFP or STm ΔssrAB + pPtrc-ssrBD56E-2HA + pPssaG-GFP for 16 h. Presence of HA tag in intracellular bacteria was examined using immunoblotting and anti-DnaK (Salmonella) antibody was used for loading control. Representative of 3 independent experiments is shown. (TIFF) [file ppat.1013728.s004.tiff]

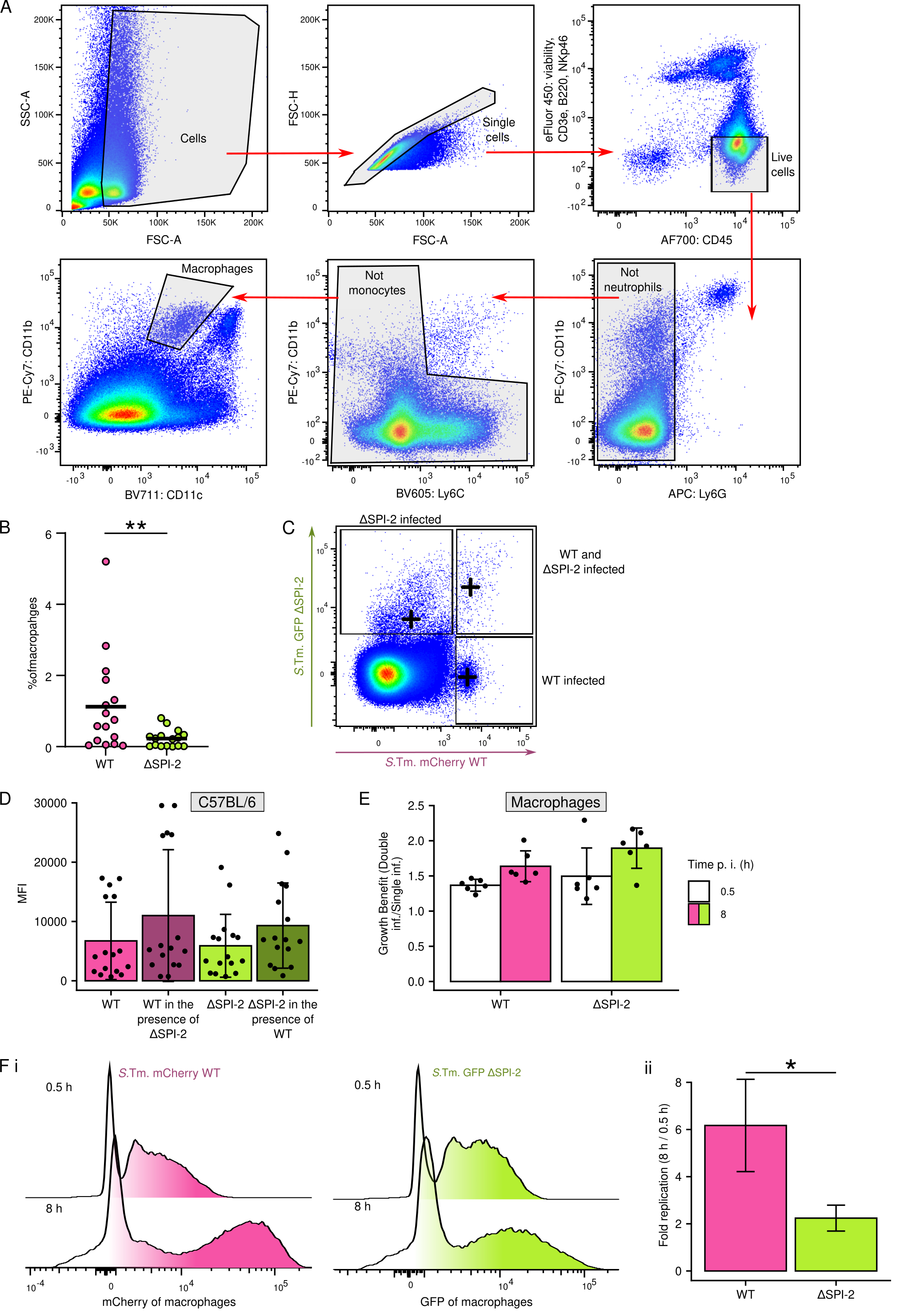

Supplement: S5 Fig — (A) Gating strategy for the detection of macrophages in spleen homogenates from C57BL/6 mice infected by STm glmS::mCherry WT and STm glmS::GFP ΔSPI-2 using flow cytometry. (B) STm glmS::GFP ΔSPI-2 has a significant survival defect in macrophages in vivo. Percentage of C57BL/6 splenic macrophages infected with by STm glmS::mCherry WT or STm glmS::GFP ΔSPI-2. **p < 0.01 (Paired two-sample t-test). (C) Representative dot plot of growth benefit data shown in Fig 5B. GFP and mCherry fluorescence intensities in splenic macrophages isolated from C57BL/6 mice infected with STm glmS::mCherry WT and STm glmS::GFP ΔSPI-2. The + signs represent median of fluorescence intensity in individual gates. (D) Median fluorescence intensities (MFI) of populations depicted in C and Fig 5B. (E) Growth benefit of bacteria from co-infections of RAW264.7 macrophages. Data are calculated from Fig 5Cii as a ratio of double-infected (dark magenta and green) and single-infected cells (light magenta and green). Calculation was done on data from both 8 h p.i. and 0.5 h p.i. (F) STm glmS::GFP ΔSPI-2 has a significant growth defect in comparison to STm glmS::mCherry WT in macrophages. (i) Representative histograms showing bacterial burden in RAW264.7 macrophages from Fig 5D at 0.5 h and 8 h p.i. (ii) Fold replication values were calculated from the medians of fluorescent intensities of mCherry and GFP fluorescence respectively as the ratio of replication at 8 h to replication at 0.5 h. Data are from 3 independent experiments in technical triplicates and show means ± SD. *p < 0.05 (Paired two-sample t-test). (TIFF) [file ppat.1013728.s005.tiff]

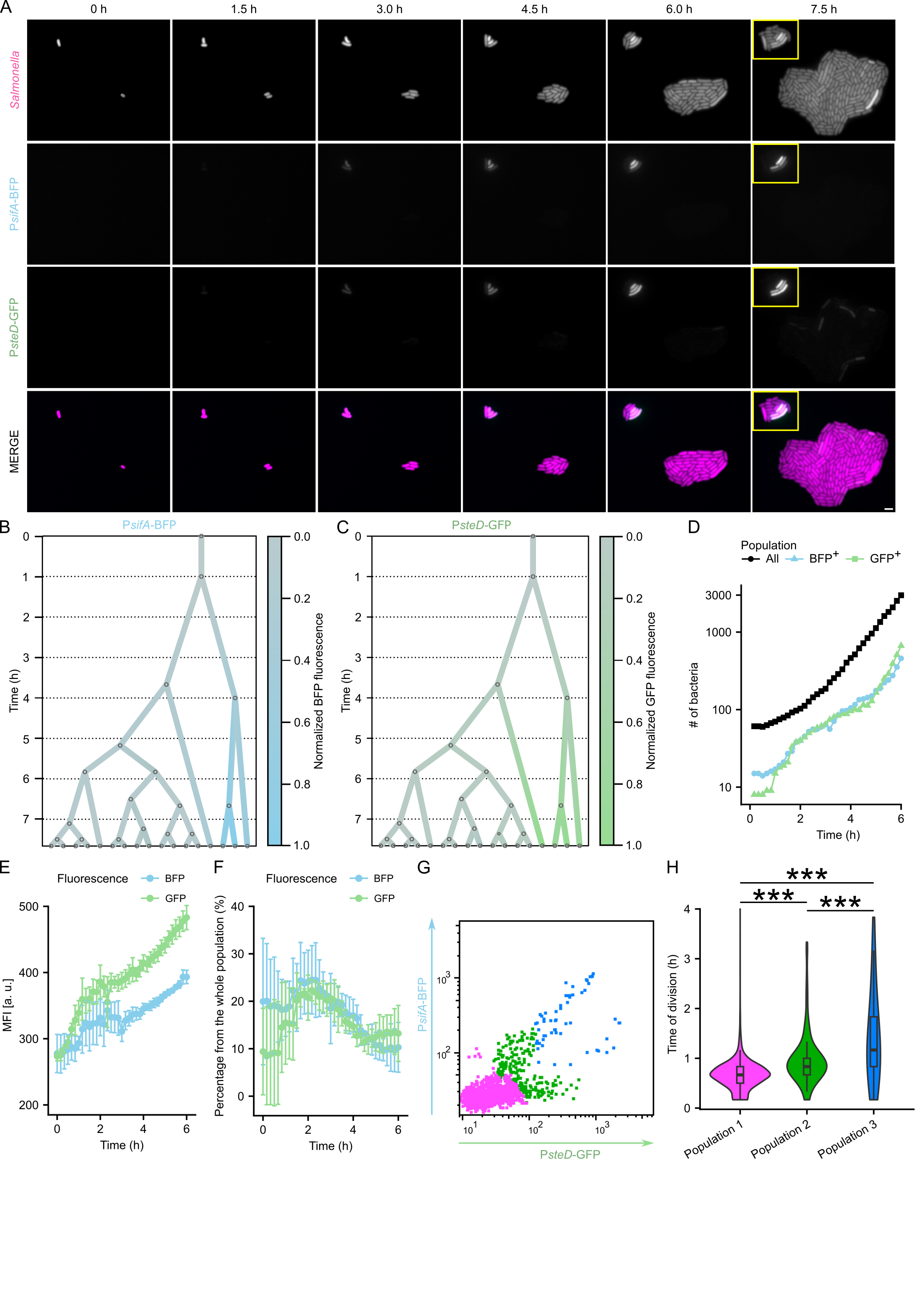

Supplement: S6 Fig — (A) Time-lapse microscopy monitoring growth of SPI-2ON Salmonella. Individual STm WT + pPsifA-BFP_PsteD-GFP were grown in MgMES pH 5 on agarose pads for 8 h. Fluorescence of BFP and GFP was monitored at 10 min intervals by fluorescence microscopy. (B and C) Lineage tree showing activity of PsifA (B) and PsteD (C) in the highlighted bacterial colony in A). Colouring of the lineage trees reflects the relative mean BFP intensity of individual cells scaled to the highest intensity in the tree. (D) The increase of the number of STm WT + pPifA-BFP_PsteD-GFP expressing or not BFP and GFP from A) over time. Data from all 3 independent experiments were pooled together. (E) Overall activity of PsifA and PsteD increases in time. The graph shows median fluorescence intensity (MFI) of BFP and GFP fluorescence of all bacteria per time point of the experiment in A). Means ± SD of data from all 3 independent experiments are shown. (F) Percentage of fluorescent bacteria from A) over time. Means ± SD of data from all 3 independent experiments are shown. (G) Dot plot showing fluorescence intensities of individual bacteria before division or at the end of the experiment. The bacteria were automatically gated into 3 populations by the FlowSOM software plugin based on fluorescent intensities. Data are from 3 independent experiments. (H) Doubling time of individual bacteria in populations shown in G). ***p < 0.001 (One-way ANOVA with Tukey HSD test). (TIFF) [file ppat.1013728.s006.tiff]

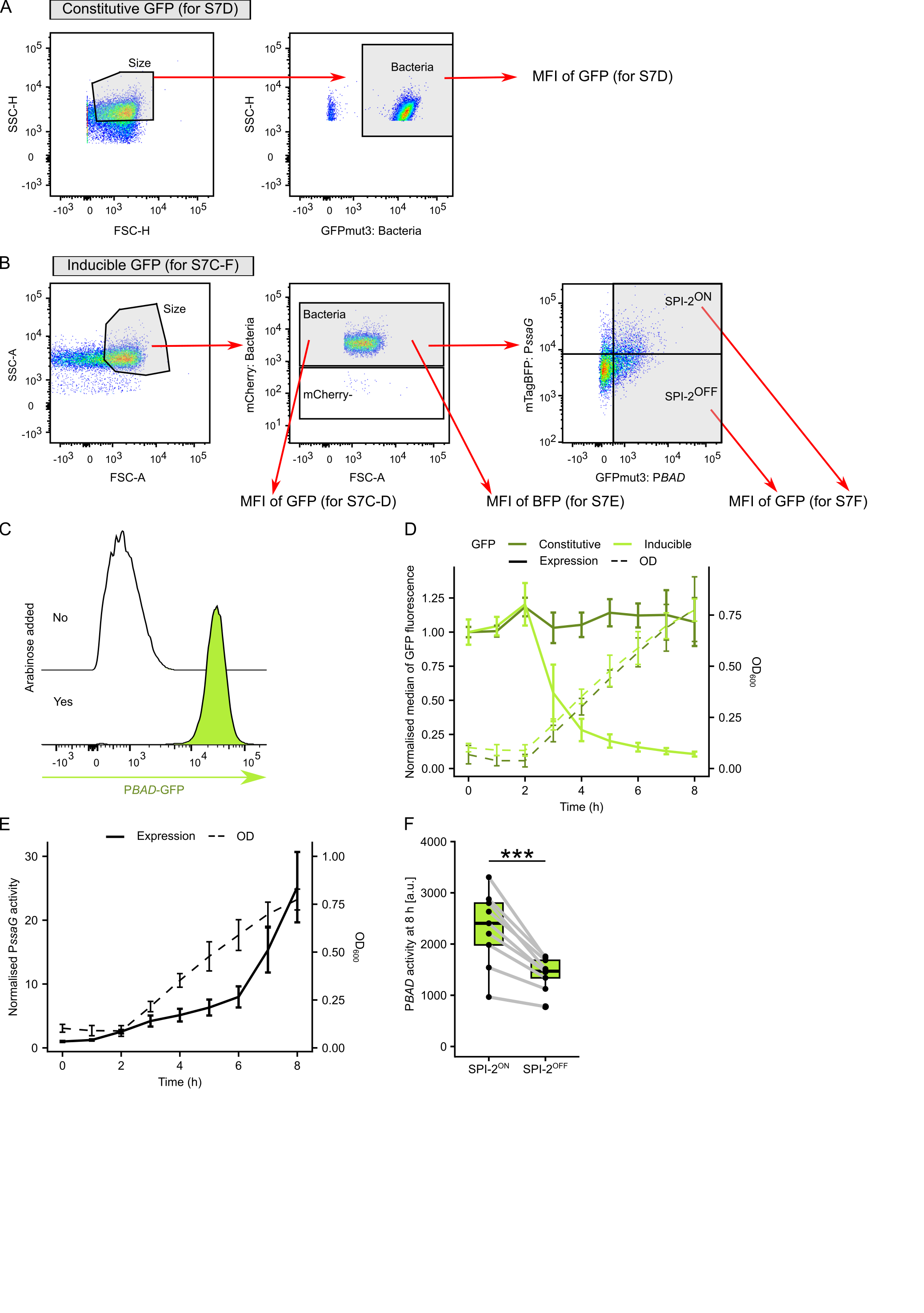

Supplement: S7 Fig — (A) Gating strategy for detection of GFP by flow cytometry in STm glmS::Ptrc-GFP (Constitutive) bacteria. (B) Gating strategy for detection of GFP and BFP by flow cytometry in STm glmS::Ptrc-mCherry + pPssaG-BFP_PBAD-GFP (Inducible) bacteria. (C) GFP expression from an inducible promoter. STm + pPssaG-BFP_PBAD-GFP was cultured in the presence or absence of 0.5% arabinose to induce uniform GFP expression. (D) STm glmS::Ptrc-GFP (Constitutive) grown in the presence of arabinose or arabinose-induced STm + pPssaG-BFP_PBAD-GFP (Inducible) were subcultured into MgMES pH 5 and GFP fluorescence (solid line, main y axis) and OD600 (dashed line, secondary y axis) were measured. at indicated time. GFP signal was normalized to the initial time point (0 h after inoculation) of each experiment. Data are from 3 independent experiments in technical triplicates and show mean ± SD. (E) PssaG activity of STm + pPssaG-BFP_PBAD-GFP (Inducible) from D). BFP was normalized to the initial time point (0 h after inoculation) of each experiment. Normalized BFP fluorescence (solid line, main y axis) and OD600 (dashed line, secondary y axis) are from 3 independent experiments in technical triplicates and show mean ± SD. (F) PBAD activity in the SPI-2ON and SPI-2OFF populations of pPssaG-BFP_PBAD-GFP after 8 h in MgMES pH 5. Data are from 3 independent experiments in technical triplicates and show mean, Q1, Q3 and grey lines connecting replications. ***p < 0.001 (Paired two-sample t-test). (TIFF) [file ppat.1013728.s007.tiff]

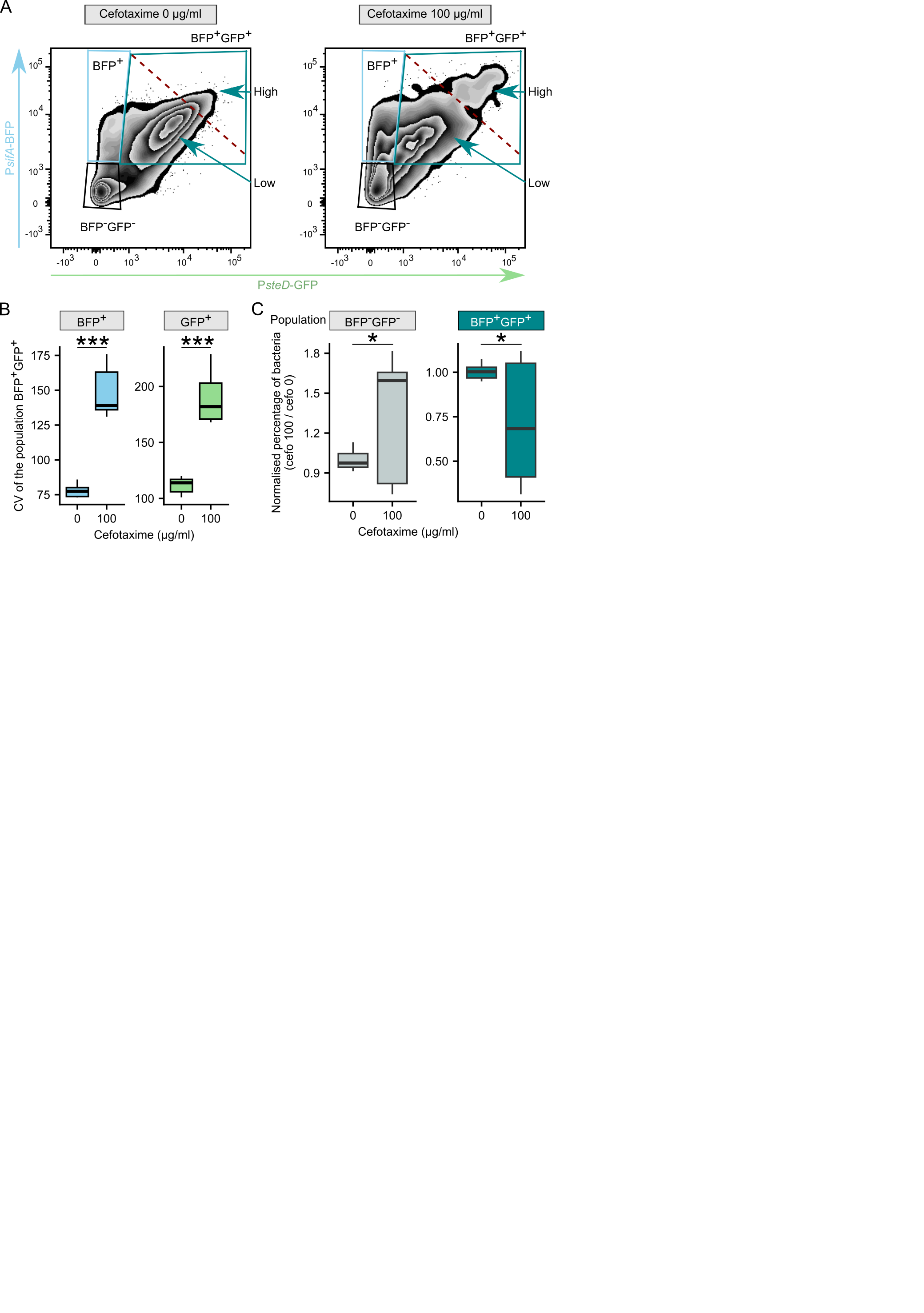

Supplement: S8 Fig — (A) Bimodality of PsifA and PsteD activity in persisters in macrophages. RAW264.7 macrophages were infected with STm WT + pPsifA-BFP_PsteD-GFP. The extracellular and intracellular proliferating bacteria were killed using cefotaxime. Gentamicin killing only the extracellular bacteria was used as a control. The host cells were lysed by hypotonic lysis 16 h p.i. and PsifA and PsteD activity was monitored as BFP and GFP fluorescence respectively in bacteria from cell lysate by flow cytometry. (B) The SPI-2ON population shows more heterogeneous activity of PsifA and PsteD in persisters. The box-and-whisker plot represents the quantification of Coefficient of Variation (CV) of BFP and GFP fluorescence in bacteria in the gate BFP+GFP+ depicted in A. Data are from 3 independent experiments in technical triplicates and show medians, Q1 and Q3. ***p < 0.001 (Two-sample t-test). (C) Activity of PsifA and PsteD in persisters differs from growing bacteria. The box-and-whisker plot represents the percentage of bacteria in BFP-GFP- and BFP+GFP+ gates depicted in A. Data are from 3 independent experiments in technical triplicates and show medians, Q1 and Q3. *p < 0.05; **p < 0.01; ***p < 0.001 (Two-sample t-test). (TIFF) [file ppat.1013728.s008.tiff]

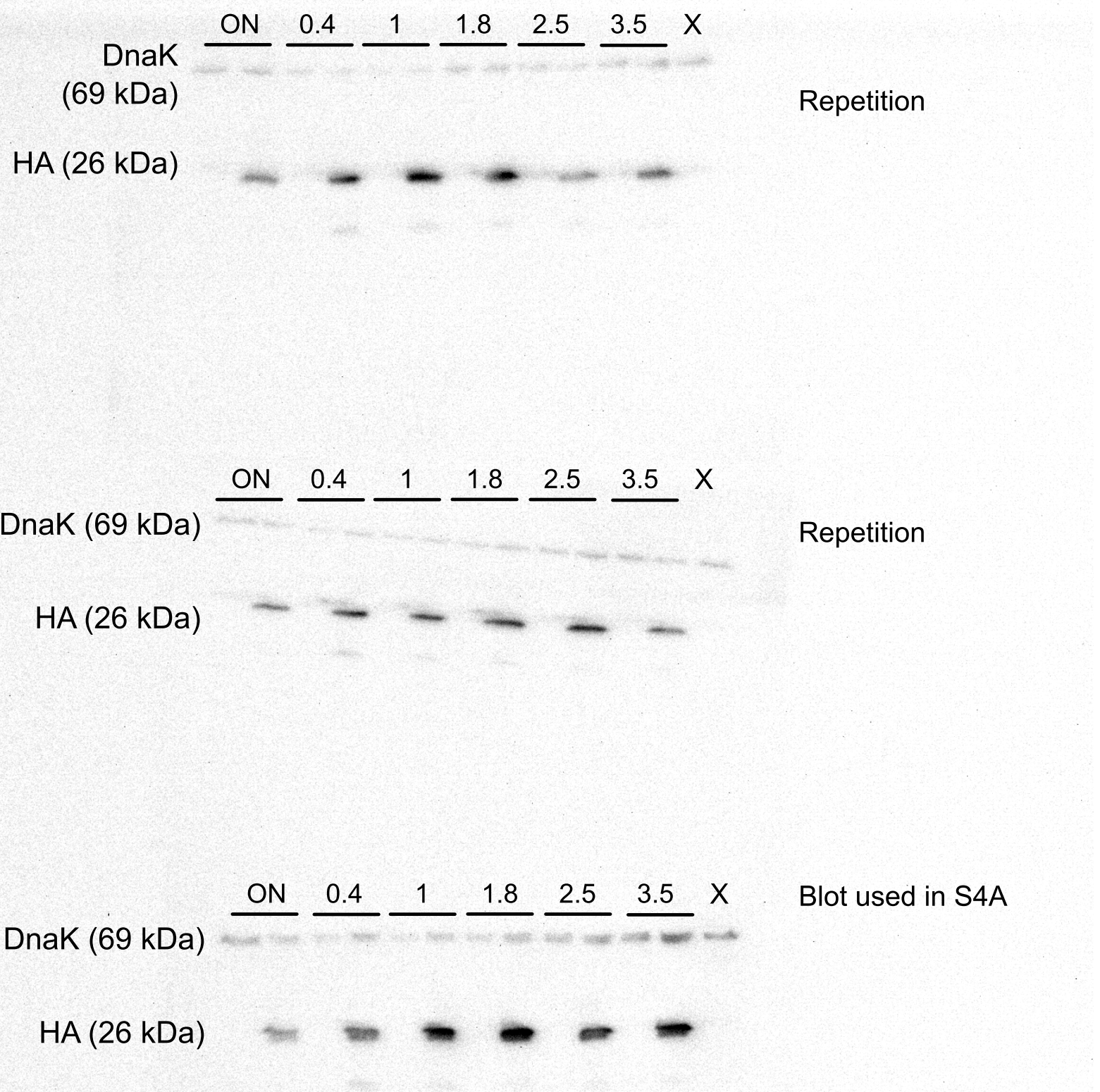

S4B

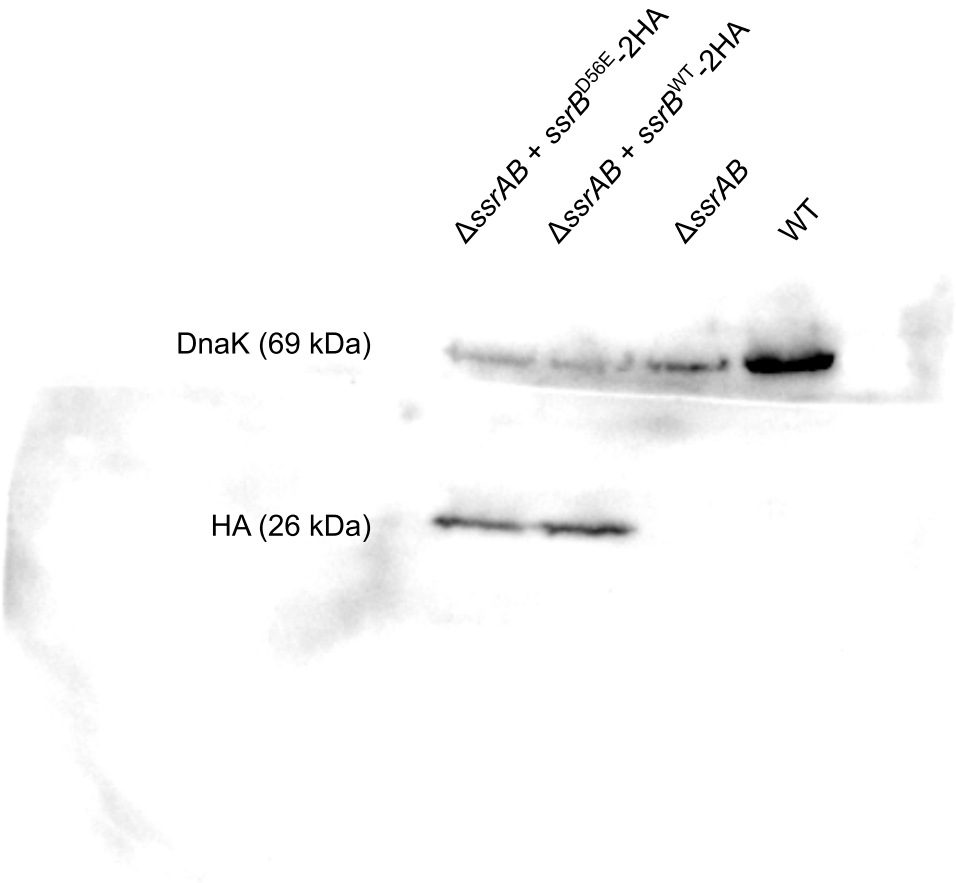

Supplement: S9 Fig — (PDF) [file ppat.1013728.s009.pdf]
