# Supplementary material for: Bimodal expression of Type 3 Secretion System 2 enables cooperative virulence among intracellular Salmonella Typhimurium
Source: PLoS Pathog. 2025 Dec 1;21(12):e1013728. doi: 10.1371/journal.ppat.1013728 (PMC12677785; doi:10.1371/journal.ppat.1013728)
Supplement: Attachment — Submitted filename: Kambova et al_rebuttal letter.docx [file ppat.1013728.s015.docx]

**Editorial office PLOS Pathogens**

**Bimodal Expression of Type 3 Secretion System 2 Enables Cooperative Virulence among Intracellular *Salmonella* Typhimurium (PPATHOGENS-D-25-01347)**

31^st^ August 2021

Dear Editor,

Many thanks for the Reviewers’ comments on the above paper and for giving us the opportunity to send you a revised manuscript. Below we highlight the criticisms of the Reviewers and provide our responses:

**Reviewer #1:**

In this manuscript by Kambova et al., the authors investigate how bimodal expression of Salmonella pathogenicity island 2 type 3 secretion systems (SPI-2 T3SS) is regulated and its influence on the intracellular survival/replication of Salmonella enterica serovar Typhimurium. In agreement with previous studies, they demonstrate that SPI-2 T3SS expression is bimodal among intracellular bacteria using flow cytometry and microscopy. The authors extend on these previous works to reveal bimodal expression of effectors as well and the ability of SPI-2 T3SS+/effector+ bacteria to support intracellular replication and subsequent escape of SPI-2 T3SS-/effector- bacteria within the same host cell. The writing throughout most of the paper is clear and detailed, with a few areas that could be expanded upon. Additionally, there are a few suggestions, including important controls, that the authors should perform to make their claims stronger.

1) *In Fig 1B&C, the authors characterize the functionality of their SsaG GFP reporter systems via microscopy and claim that the “majority of bacteria from both reporter strains showed high GFP fluorescence” (line 90-91). However, based on images presented, it looks like less than half of the bacteria are expressing GFP and not all the non-GFP-expressing bacteria are marked with a pink arrow. Can the percentage of GFP expressing bacteria be quantified from these images or the claim adjusted with arrows highlighting all non-GFP-expressing bacteria? However, it is clear from the flow cytometry done in Figure 1D, that a majority of the bacteria are indeed expressing GFP.*

**Response:** We have quantified the percentage of GFP-expressing bacteria from both reporter strains. This is now stated on line 90 of the manuscript with highlighted changes. Approximately 66.9 ± 14 % of *Salmonella* with the plasmid reporter system and 67.4 ± 12 % of *Salmonella* with the chromosomal reporter expressed GFP under used conditions. However, some bacteria show borderline levels of GFP fluorescence signal and this makes similar quantification less reliable than flow cytometry showing clear separation of GFP^+^ and GFP^-^ populations. In the Fig 1B and C, we marked only bacteria that show GFP fluorescence signal below a threshold setup using a reporterless strain.

2) *As a control, can bimodal expression of SsaG, SifA, and SteD also be seen via qPCR in addition to promoter-linked GFP/BFP expression? It’s important that their reporter system accurately reflects the true expression of these proteins.*

**Response:** We have not included these experiments into the original manuscript since we faced high cross-contamination between individual sorted populations (i.e. SPI-2^ON^ and SPI-2^OFF^) that reached up to 25% in some experiments. This led to high inter-experiment variability and decreased ΔΔCt ratios in some experiments reflected as higher than expected overall p-value (one sample T-test). We show the obtained results here for reference. The dashed line marks a level usually considered for a significant change.

3) *In Fig 1E, Fig S1A, and Fig S1G-J, the percentage of SsaG-, SifA-, and SteD- bacteria decreases over time up to 5-6 hours in MgM-MES pH 5.0. The percentage of SsaG-, SifA-, and SteD- bacteria (represented by GFP/BFP expression) at the end of the kinetic experiment are quite low. Are these populations sustained and significant? Or are SsaG and effector expression temporal rather than consistently bimodal? The following are possible ways of validating:*

*a. Extending the kinetic experiment to longer time points would help to confirm that these populations are always present and the decrease in their abundance plateaus such as in Fig S1H and S1J.*

**Response:** In the original version of the graph in Fig. 1E ii, we used the x-axis with constant distance between individual data points instead of showing real time differences between individual time points. We have now corrected this issue and apologize for the confusion. The current version of the graph shows clearly that the abundance of the individual populations reaches a plateau at approximately 2 h and stays more or less constant until 5 h. In our initial experiment, we also included time points between 2 and 6 h after inoculation, but since these data did not bring any new information, we did not include them in the repeated experiments. Below we attach the graph with time points not included in the manuscript.)

*b. Placing GFP/BFP expression under a constitutive promoter can act as a positive control whereby there should be no GFP-/BFP- populations and demonstrate these low percentages of SsaG-, SifA-, and SteD- bacteria are significant and not an artifact of flow cytometry gating or using GFP/BFP as a proxy for the expression of these proteins. For example, in Fig 6A, it looks like there is also a small GFP- population even under the pBAD promoter.*

**Response:** We constructed *Salmonella* mCherry + pP*rpsM*-GFP expressing mCherry under the control of the constitutive *trc* promoter as we use in the manuscript, and GFP under the control of the ribosomal *rpsM* promoter. Only 0.054 % of mCherry^+^ events did not show GFP fluorescence. This is about two orders of magnitude less than the amount of GFP^-^ events observed with our P*ssaG*-GFP reporters suggesting that data processing is not responsible for the appearance of the non-expressing population. The mCherry^-^GFP^-^ events shown here are probably non-fluorescent particles originating from sheath fluid.

To avoid confusion originating from the small peak of GFP^-^ population in Fig 6A, we have replaced this figure with data from a different replicate of the experiment.

4) *In Fig 3, do the sorted individual subpopulations after the first infection establish the other populations during axenic regrowth to exponential phase in LB or only after infecting Mel JuSo cells? Flow cytometry of the inoculum immediately after sorting and after regrowth prior to second infection would clarify.*

**Response:** After regrowth prior to the second infection, neither of the cultures show significant expression of the reporters. Example data from samples before sorting, reanalysis after sorting, and after regrowth of individual sorted populations is shown below. The subpopulations after the initial sorting are shown in Fig. 3B. These data clearly show that the reestablishment of the expressing populations is a process that follows reinfection.

5) *In addition, none of the calculations in this paper take into account the half-life of GFP or BFP. How do these effect the interpretation of all their results?*

**Response:** The half-life of both GFPmut3 and mTagBFP2 used in our experiments is longer than duration of the experiment (i.e. > 24 h for GFPmut3 and ~ 88 h for mTagBFP2; Andersen JB, *et al 1998*, DOI: 10.1128/aem.64.6.2240-2246.1998; Subach OM, *et. al. 2011*, DOI: 10.1371/journal.pone.0028674). Therefore, our results reflect expression at any time since the beginning of the experiment, rather than the actual state at the end of the experiment. The subpopulation expressing the studied virulence factors at any specific time would therefore be lower. To reflect this, we have used destabilzed GFP version (GFP-ASV) in Fig. 1H and I (see also the Reviewer’s 3 comment #1).

6) *In Figure 5B and 5Cii, only a ratio of “Double inf. / Single inf.” is shown. Displaying the raw MFI values in individual subpanels, without the ratio, in singly versus doubly infected macrophages for both WT mCherry and ΔSPI-2 GFP would be helpful to emphasize the claim that SPI-2OFF benefit from the effectors of SPI-2ON. The raw data before calculating the ratio should at least be in the supplemental figures.*

**Response:** Raw data (MFI) for Fig 5B are now in S5D Fig. Raw data for Fig 5C (now Fig 5Cii) are in Fig 5Ci and intermediate data for growth benefit of coinfections (i.e. not normalised to 0.5 h p. i.) in S5E Fig.

7) *In Figure 5Dii, showing the raw values would be helpful to assess the heterogeneity per condition and the magnitude of difference since the difference in the median values of the violin plots seem minor.*

**Response:** Thank you for this suggestion. We have replaced the violin plot with a dotplot in the manuscript. Raw data for this experiment are available at Zenodo (DOI: 10.5281/zenodo.17199173) in file F5_data, sheets F5D-WT and F5D-SSAV.

8) *In lines 295-298, more description of how bacterial subpopulations are identified and how doubling time is determined will be beneficial. What features led the algorithm to separate out each subpopulation? Are there enough individual bacteria in Population 3 to form its own population?*

**Response:** The algorithm clustered the analyzed events (i.e. bacteria tracked from their appearance as a new daughter cell until the consecutive division) based on the fluorescence of both mTagBFP2 and GFPmut3. Only fluorescence signal was used for the clustering. We have described this in the Methods section on lines 565 to 568 (manuscript with highlighted changes) now.

Population 3 contains more than 500 events that corresponds to approximately 5.6 % of all analyzed events.

9) *The captions for Fig S7 need to be fixed. The text does not align with Fig S7.*

**Response:** We apologize for the oversight. The captions were corrected.

*a. It is not clear how GFP fluorescence and PssaG activity are normalized.*

**Response:** We have clarified the normalization in the figure legend now. Briefly, GFP fluorescence was normalized to the initial point (0 h after inoculation). P*ssaG* activity was normalized in a similar way.

*b. Fig S7D is important for the claim that SPI-2ON bacteria replicate slower than SPI-2OFF via GFP dilution. However, it is not clear based on the main text and the captions of the panel how this experiment was done. Fixing the captions and providing more information in the main text (ie time point, whether FACS sorting was involved, etc) will help. It seems like Fig S7D is currently linked with the caption for Fig S7F; if so, how were the quadrants defined? Gating strategy should be shown.*

**Response:** In S7 Fig, *Salmonella glmS*::Ptrc-GFP (Constitutive) and *Salmonella* WT + pP*ssaG*-BFP_P*BAD*-GFP (Inducible) were grown in LB in the presence or absence of arabinose until late stationary phase before subculturing into MgMES pH5.0 without arabinose to allow dilution of the pre-synthesized GFP. OD_600_, and GFP and BFP fluorescence was measured at indicated time points. The gating strategy describing data processing at individual panels was added as current S7A and S7B Figs. All flow cytometry data were acquired by a conventional flow cytometer. Bacteria were identified based on the size and GFP (*Salmonella glmS*::P*trc*-GFP) or mCherry (*Salmonella* WT + pP*ssaG*-BFP_P*BAD*-GFP) fluorescence. MFI of GFP and BFP was used to control for arabinose induction efficiency (S7C Fig), to analyse GFP dilution (S7D Fig), and overall SPI-2 expression in induced *Salmonella* WT + pP*ssaG*-BFP_P*BAD*-GFP (S7E Fig). SPI-2^ON^ and SPI-2^OFF^ *Salmonella* WT + pP*ssaG*-BFP_P*BAD*-GFP still showing GFP fluorescence were identified based on BFP fluorescence 8 h post subculturing and their remaining GFP levels were analysed to compare proliferation speed in the subpopulations. This is now more carefully described in the main text and in the corrected caption of S7 Fig.

10) *For the GFP dilution infection experiments described in Fig 6, is the presence of L-arabinose eliminated prior to infection with macrophages? This should be clarified in the text and Fig 6A schematic as it affects how the data is interpreted.*

**Response:** Arabinose was added into the bacterial growth medium only. The infection was carried out in the absence of arabinose. We have explained this in the main text and corrected the schematics in Fig. 6A accordingly now.

*a. The reason why the four separate populations are defined is not clear in the text. If the goal is to compare the proliferation rates between SPI-2ON and SPI-2OFF, GFP MFI of SsaG+ (BFP+) vs SsaG- (BFP-) would be a more direct comparison instead of comparing SsaG+ (BFP+) separately with GFP+ and GFP- BFP- as shown.*

**Response:** The SPI-2^OFF^ (BFP^-^) subpopulation is effectively divided into two parts – GFP^-^BFP^-^ proliferating and GFP^+^ non-proliferating. Since the GFP^+^ population does not proliferate, it cannot be used to estimate proliferation speed, and (technically) averaging the MFI of the GFP^-^BFP^-^ and GFP^+^ parts would artificially hide possible differences. Instead, we used the GFP^+^ subpopulation as an intrinsic standard and compared the GFP^-^BFP^-^ and BFP^+^ subpopulations first to compare the proliferation speed of GFP^-^BFP^-^ and SPI-2^ON^ *Salmonella* respectively (panel *ii*). Further, we examined whether there is a difference between faster and slower proliferating SPI-2^ON^ bacteria in the activity of the SPI-2 expression (panel *ii*). This is now described in the text.

*b. To quantify GFP dilution as a proxy for replication rate, the GFP MFI between two time points as a “fold replication” is helpful ie between 2 hrs and 6/8 hrs (Figueira et al. mBio 2013 & Hamblin et al. mBio 2023). Here, the authors are comparing GFP MFI of a single time point without normalizing to where the initial GFP MFI starts at upon SsaG formation.*

**Response:** Figueira *et al*. (2013) and Hamblin *et al*. (2023) compare the “fold replication” between individual bacterial strains that might have different starting GFP MFI, therefore normalization to the starting time point is crucial. In our experiment, we compare bacteria of the same strain grown in a single culture infecting host cells (Mel JuSo) in the same sample. All these bacteria have uniform starting GFP MFI. At 2 h post infection, we still do not see significant separation of individual bacterial subpopulations based on the GFP level (please see the figure below). This happens first at 4 h p.i., a time point too close to the final sample collection to be useful for normalization. Therefore, we believe that any normalization here would only hide the raw data and would be in contrast to the comments number 6 and 7 here.

*c. Minor note, it would be more consistent to provide one label instead of switching between T3SS-2ON and SPI-2ON.*

**Response:** We apologize for this oversight. We unified it to SPI-2^ON^ in the new version of the manuscript.

11) *In Fig 6E, there isn’t statistical significance, so the claim in lines 339-340 of a “significant increase” needs to be changed.*

**Response:** Since the first submission of the manuscript, we have done several other infection experiments with the same reporter system. The data obtained was added to the Fig. 6E. Also, we included an additional data point (10 h p.i.). The current p value shows statistical significance between 8 and 16 h p.i. As we have less data for 10 h p.i., we can only assume a trend, but this did not reach a statistical significance.

12) *In Fig 7, the authors claim that extracellular bacteria have a higher proportion of SPI-2OFF than intracellular bacteria. A key assumption is that upon inducing host cell lysis, the expression of SsaG isn’t downregulated from SPI-2ON bacteria once outside of the nutrient-limited SCV and in the extracellular media. Control experiments are needed ie comparing SsaG expression after transferring bacteria from MgM-MES pH 5 media to host cell media in the time scale of the infection/lysis experiments.*

**Response:** In this experiment, we used fluorescence of GFPmut3 expressed under the control of P*ssaG* as a proxy for *ssaG* expression. However, the GFPmut3 has half-life approximately 24 h and thus represents the expression of *ssaG* by the GFP^+^ bacteria at any time during our experiment. Therefore, downregulation of *ssaG* expression after egress of the GFP^+^ bacteria would not significantly influence GFP fluorescence. Moreover, the host cell media contains gentamicin (20 µg/ml), which kills the escaped bacteria prohibiting dilution of GFP. However, we measured the stability of GFP fluorescence after shift from the MgMES pH 5.0 to DMEM + 10 % FCS + 20 µg/ml gentamicin and did not observe any loss of GFP fluorescence (please see below).

13) *In line 426, it’s worth specifying that only SteD and SifA were tested in this work in the context of persisters. Would some persisters also not express other effectors?*

**Response:** We have modified the Discussion section on current line 452 (manuscript with highlighted changes) to be more specific. Regarding other effectors, we assume that some of the SifA^+^SteD^+^ persisters would not express some of the remaining SPI-2 T3SS effectors, while some of the SifA^-^SteD^-^ persisters might express some other SPI-2 T3SS effector.

14) *In the Discussion, perhaps the authors can discuss why SifA+ SteD- or SifA- SteD+ populations present in vitro (Fig 2E) are not present upon in vivo infection in C57BL/6 mice (Fig 2F).*

**Response:** We have added a paragraph discussing this on lines 388 to 400 in Discussion (manuscript with highlighted changes).

15) *Very minor but in line 979, “analyzed” is used while “analysed” was used throughout the rest of the text.*

**Response:** We thank the Reviewer for pointing out this oversight. It was corrected in the current version of the manuscript.

**Reviewer #2:**

The manuscript presents a compelling study on the bimodal expression of SPI-2 T3SS and its effectors in Salmonella Typhimurium, exploring its physiological significance during infection. While the work is interesting and potentially impactful, several critical technical and presentation issues need to be addressed to enhance the clarity, rigor, and reproducibility of the findings.

**Major Issues:**

1) *The authors should clarify whether the constructed fluorescent reporter plasmids are stably maintained during in vitro and in vivo infections. What is the loss rate of the plasmid in host cells or mice? How might plasmid loss affect the quantification and interpretation of SPI-2ON versus SPI-2OFF populations? Additionally, the authors should describe in detail the procedures for recovering bacteria from lysed cells or mouse organs and whether these steps could artificially alter SPI-2 expression.*

**Response:** In our preliminary experiments, we focused on plasmid maintenance by bacteria throughout the experiments. We observed no plasmid loss during either the *in vitro* experiments or during the first week of *in vivo* experiments. We did not include the longer time points, where we had observed plasmid loss, in the manuscript. This is now mentioned in the manuscript on lines 523 to 525 (manuscript with highlighted changes).

In the case of cell cultures, the bacteria were recovered from cells lysed using sterile ddH_2_O following 3 washing steps with sterile PBS. After mixing the lysate, bacteria were fixed using 4% PFA for 10 min at 37°C, washed with PBS and analyzed by flow cytometry. This is described in the methods section on lines 503 to 505 (manuscript with highlighted changes). Our preliminary experiments showed that this manipulation does not influence the percentage of GFP^+^ bacteria.

To analyze bacteria recovered from host tissues, these were dissociated in RPMI containing collagenase IV (1 mg/ml), DNAse I (0.1 mg/ml), and 2% FCS for 30 min at 37°C. The cell suspension was filtered through a 70 µm cell strainer on ice and washed twice with Ca^2+^, Mg^2+^ free HBSS supplemented with 10 mM HEPES and 2% FCS. After this step, the individual cells were processed as cell cultures described above. This is described in the methods section on lines 518 to 523 (manuscript with highlighted changes).

2) *Figure 1F and 1G, given that Salmonella expresses red fluorescence and ssaG+ bacteria express green fluorescence, co-localization should appear yellow. However, no yellow fluorescence is visible in the merged images.*

**Response:** To make our figures colorblind-friendly, we used magenta instead of red in the manuscript. In this setup, colocalization appears as white. To make colocalization yellow, we show *Salmonella* in the red coloring here (yellow arrowheads pointing towards red *Salmonella* expressing GFP, while the red arrowheads pointing towards red *Salmonella* not expressing GFP).

**Minor Issues:**

1) *The schematic in Figure 1A is unclear. The two bacterial strains used should be explicitly labeled as: S. Typhimurium 12023 glmS::Ptrc-mCherry + pPssaG-GFP and S. Typhimurium 12023 glmS::PssaG-GFP. In Figure 1B, it is not clear whether the red fluorescent protein is being correctly expressed. In Figure 1D, the peaks must be annotated for clarity, and the figure legends need clarify what is MES.*

**Response:** The labeling in Fig 1A has been corrected. In Fig 1B, we omitted the red fluorescent signal and are only showing the Phase contrast and GFP fluorescence for the space constraint reasons and because the mCherry signal masks the GFP signal in the merge, For comparison and to show that all bacteria indeed express mCherry, we include the full figure here. Fig 1D annotation has been modified.

2) *Lines 199 and 336, the authors mention that bacteria are "dead." What experimental evidence supports this claim?*

**Response:** In both cases, the claims are speculations trying to explain why the bacteria in the experiments did not respond to the treatment. In the first case (current line 206 of the manuscript with highlighted changes, S3A Fig.), about 60% intracellular SPI-2^OFF^ bacteria did not respond to arabinose. This could be explained by either arabinose not reaching bacteria in some SCVs or these bacteria could be dead. In the second case (current line 346 of the manuscript with highlighted changes, Fig 6B to D), a subpopulation of bacteria within host cells neither expressed SPI-2 (increase in BFP fluorescence) nor proliferated (decrease in GFP fluorescence). This could be explained by either entering a non-proliferating state (e.g. persisters) or being killed by the host after infection. In either case, we did not follow these bacteria further and therefore did not characterize them in more detail.

3) *The rationale for choosing Mel JuSo cells for the infection experiments should be explained. What are the unique features of this cell line that make it suitable for this study?*

**Response:** In contrast to other routinely used epithelial cells such as HeLa, we have not observed hyperproliferation of cytosolic *Salmonella* in Mel JuSo cells, that could artificially increase the proportion of the SPI-2^OFF^ subpopulation. This is now described in the manuscript on lines 486 to 489 (manuscript with highlighted changes).

4) *Why did the authors choose to heterologously express LAMP1-mTurquoise rather than use endogenously expressed lysosomal markers for SCV?*

**Response:** We tested anti-LAMP1 antibodies from Invitrogen (cat. n. PA1-654A), Cell Signaling Technology (cat. n. 9091), and AbCam (cat. n. AB320851) using either a PFA, methanol, or glutaraldehyde fixation protocols. However, these antibodies had too high background to allow reliable detection of SCV.

5) *It would be clearer if Figure 4ii is presented with strain names on the x-axis and GFP fluorescence intensity on the y-axis, which would improve readability for the audience.*

**Response:** We understand the rationale behind this suggestion. However, we chose the horizontal orientation of the graphs in panels (ii) in Fig. 4 to align the graphs with the corresponding histograms in panels (i).

6) *The SPI-2OFF peak in Figure 4B is noticeably higher than in Figures 1D. This inconsistency should be explained or discussed.*

**Response:** Fig. 1D represents experiments in minimal media with negligible autofluorescence from other particles present in the experiment. In contrast, Fig. 4B represents data from host cell lysate that contains highly autofluorescent phagosomes and endosomes. Therefore, the background must be adjusted to reflect this. Similar adjustment in minimal medium vs. host cell lysate can be seen in other figures such as Fig. 4A (minimal medium) vs. 4C (host cell lysate), that correspond to similar experimental set up throughput the article. This does not influence data interpretation as we do not compare the MFI of individual peaks between different experimental setups.

7) *The statistical comparisons in Figures 5B and 5Cii are not well explained. Which groups are being compared? Please clearly describe the comparisons and significance testing used.*

**Response:** In both graphs, we compare the growth benefit of both strains to a hypothetical value of no benefit (One-sample t-test, hypothesized population mean (μ) = 1, significance levels labelled with stars) as well as between both strains (paired two-sample t-test, significance levels labelled with hashtags). To make the statistics clearer, we have graphically separated the two scenarios more clearly and described in the figure caption now.

8) *In Figure 5D, why is the bacterial burden of WT in singly infected cells significantly lower than in co-infected cells? Please clarify.*

**Response:** This is an interesting question that we have not been able to address to our satisfaction yet. At the moment, we can only speculate that increasing the amount of the SPI-2^OFF^ bacteria leads to splitting of the bactericidal capacity of the infected macrophage among more bacteria thus effectively decreasing the amount bactericidal compounds reaching the WT-containing SCVs. We plan to test this hypothesis after we analyze the signaling pathways regulating SPI-2^ON^/SPI-2^OFF^ balance in more detail in our future work.

9) *Figures 6B and 6C report data from 6 hours and 8 hours post-infection, whereas Figure 6E presents 8 and 16 h time points. Please explain the rationale behind using different time windows.*

**Response:** The time points in Fig. 6B and C were selected to detect GFP dilution before the GFP signal drops too low. The time points in Fig. 6E reflect the earliest (selected for host cell death, as in Fig. 7B) and the latest time point used throughout the article. Since the experiments in Fig. 6B and C use a different reporter system from those in Fig. 6E, we do not think we can combine their outcomes in the same figure.

10) *Why was cefotaxime used to remove extracellular and intracellular bacteria in Figure S8? Is there published evidence supporting the use of this antibiotic for such purposes?*

**Response:** While gentamicin, used in most of our experiments, kills the extracellular bacteria only, cefotaxime kills proliferating bacteria and can permeate host cell membranes in quantities sufficient to kill not only the extracellular but also the intracellular proliferating bacteria. In the experiments reported in S8 Fig., we used protocols similar to that described in Ekinci *et al*., 2002 (DOI: 10.1046/j.1469-0691.2002.00457.x), Helaine *et al*., 2014 (DOI: 10.1126/science.1244705), and Stapels *et al.*, 2018 (DOI: 10.1126/science.aat7148).

11) *In line 371, the sentence stating, "The SPI-2OFF Salmonella proliferated more rapidly and egressed more quickly from infected host cells" is misleading. Since cell lysis releases both SPI-2ON and SPI-2OFF populations, this needs to be more accurately stated.*

**Response:** This sentence was stated more accurately on lines 381 (manuscript with highlighted changes) now.

12) *Dynamics of SPI-2ON/OFF Ratios, could the authors provide additional time points showing the dynamic changes in the ratio of SPI-2ON and SPI-2OFF bacteria during infection? Is there a consistent trend of SPI-2OFF enrichment in later stages?*

**Response:** We have added time point 10 h p.i. into the graph in Fig. 6E and more repetitions for 16 h p. i.. Although the only significant difference is between early (8 h p.i.) and late (16 h p.i.) time points, this is due to a big difference in number of repetitions, and a trend is apparent with the amount of the SPI-2^OFF^ bacteria increasing with time.

13) *In lines 390–391, the authors claim regulation occurs at two levels. Please specify these layers explicitly. From the data, only SsrA-SsrB regulation is apparent. Is there additional evidence to support the second regulatory layer?*

**Response:** The first and crucial level of regulation comes from the SsrA-SsrB interaction, while the second layer is probably represented by heterogeneity among individual SCVs. This is stated more explicitly on lines 414 to 424 (manuscript with highlighted changes) now.

14) *In the Discussion section, please refer to specific figures when discussing experimental results to guide the reader more effectively.*

**Response:** We have added references to specific figures in the Discussion section.

15) *Line 866-867, Please clarify why an OD 1.8 is used to represent the late exponential phase, while a time point of 16 hours is used to represent the late stationary phase.*

**Response:** In our conditions, the bacteria reach late exponential phase at OD_600_ = 1.8. At this point, we observe high activity of SPI-1 T3SS. In contrast, at late stationary phase (16 h were selected to enable collection of the remaining samples) the SPI-1 is not expressed in our conditions, and the bacteria start expression of SPI-2 faster after shift into inducing conditions (S1A Fig. vs Fig 1D). This is stated more explicitly on lines 920 and 921 (manuscript with highlighted changes) now.

16) *Line 889, Please provide complete strain information.*

**Response:** The Fig. 5A is a schematic representing the workflow for Fig. 5B and S5A to C Fig. We have done these experiments mostly using *S*Tm *glmS*::mCherry WT and *S*Tm *glmS*::GFP ΔSPI-2, although we did color swap control experiments for all panels in Fig. 5 and S5 Fig. The strains used for results presented in the manuscript are listed in the specific panels.

17) *Line 919, (ii) changed to (iii).*

**Response:** This has been changed, thank you for pointing it out.

18) *Salmonella should be italicized throughout the manuscript. Units such as “mL” and “μL” should be standardized and formatted correctly across the text.*

**Response:** All *Salmonella* is italicized, and units are reformatted now.

19) *The manuscript requires improved writing and organization. Some paragraphs contain only a single sentence. Additionally, the references are not formatted according to the journal's guidelines.*

**Response:** We had revised the text and reformatted the references (Vancouver style) according to the journal’s guidelines.

**Reviewer #3:**

The bacterial Type III Secretion System (T3SS) encoded on the Salmonella pathogenicity island 2 (SPI-2) is a key and highly conserved virulence mechanism in many Salmonella serovars. This system enables the translocation of effector proteins from vacuole-bound intracellular bacteria into the cytosol of host cells. These effector proteins support various aspects of Salmonella pathogenesis, including vacuole integrity, intracellular nutrient acquisition, and immune evasion.

In the present manuscript, the authors report that the expression of the SPI-2 T3SS and its effector proteins follows a bimodal pattern. They present a comprehensive analysis using multiple approaches, including flow cytometry, fluorescence microscopy, and both in vitro and in vivo infection models. Their findings show that this bimodal expression is regulated by the response regulator SsrB and that the two subpopulations SPI-2 ON and SPI-2 OFF mutually support each other. Specifically, SPI-2 ON Salmonella secrete effectors that enable efficient proliferation of SPI-2 OFF bacteria within the nutrient-limited intravacuolar environment. In turn, SPI-2 OFF bacteria re-establish the bimodal expression and re-establish the SPI-2 ON sub-population during intracellular replication.

In summary, this is a nicely written manuscript which provides convincing evidence that Salmonella exhibits bimodal expression of the SPI-2 T3SS, which may constitute an adaptive division of labor strategy that enhances Salmonella fitness and dissemination during infection.

**Major Issues:**

1) *A major concern is the absence of degradation tags in the SPI-2 reporters used in this study. As a result, the data likely reflect a relatively static view of the bacterial population. SPI-2 OFF bacteria may still appear fluorescent due to previous reporter signal inherited from a SPI-2 ON parent or even grandparent cell. This lack of temporal resolution could affect the interpretation of the expression dynamics and potentially obscure the true proportions of SPI-2 ON and OFF subpopulations. To resolve this issue, the authors should repeat key reporter assays using destabilized fluorescent proteins to enable more accurate, real-time assessment of SPI-2 expression dynamics and reduce signal carryover from ancestral ON states.*

**Response:** Thank you for this important comment. We agree that comparison of stable and destabilized reporter versions helps in general. We have selected the ASV destabilization tag (Anderson *et al.,* 1998; 10.1128/aem.64.6.2240-2246.1998) for our GFP reporters and present the data in Figs. 1H, I, 2F, and S2A Fig now. Although we observed bimodality of SPI-2 T3SS as well as effectors expression, less bacteria showed the ON state using the destabilized reporter (ASV tag). Importantly, the resolution between the ON and OFF state worsened substantially using the destabilized reporter as expected, probably due to a decrease in GFP molecules present in the bacteria. This makes exact quantification of bacteria in the ON state more difficult and less reliable.

We have also tried the LVA destabilization tag (Anderson *et al.,* 1998; 10.1128/aem.64.6.2240-2246.1998) in our reporters. While the ASV-tagged GFP has half-life about 4 h, the LVA-tagged GFP has half-life only about 0.5 h. We failed to detect fluorescence of GFP-LVA in any of our experimental conditions including the minimal MgMES pH 5.0 media for either the SPI-2 T3SS or effectors, although it is well known that they are expressed in these conditions. This demonstrates that the detection limit is a crucial limitation of the destabilized reporters and we decided not to repeat further experiments using these reporters.

Importantly, we did not observe the destabilization effect of the ASV tag on mTagBFP2. Therefore, we could not directly compare individual effectors between each other and decided to show the pP*sifA*-GFP-ASV reporter only, since the pP*steD*-GFP-ASV reported behaved similarly and does not add any new information to the manuscript.

2) *Figure 7: the authors propose that SPI-2 OFF bacteria are responsible for driving escape from either macrophages or epithelial cells. However, the experimental readout lacks precision. Bacterial exit from host cells is likely asynchronous, meaning that bacteria which escape early may have had up to two hours to replicate extracellularly. During this time, the SPI-2 reporter signal could be rapidly diluted through cell division, potentially leading to an overestimation of the SPI-2 OFF population. To strengthen their conclusion, the authors could complement their escape assays with the TIMER-Bac system (Claudi et al., 2014) to infer the relative age and division history of escaped bacteria, thereby distinguishing recently egressed SPI-2 OFF cells from those that may have undergone extracellular replication and diluted their reporter signal.*

**Response:** We agree that the escape of bacteria from host cells is likely asynchronous. Therefore, in the experiments described in Fig 7, the bacteria were allowed to escape into the DMEM medium containing 20 µg/mL gentamicin. We have updated the schematic to highlight this now.

In our control experiments, we observed a fast decrease in viability of *Salmonella* after transfer of bacteria to DMEM + 10 % FCS + 20 µg/mL gentamicin (please see the results below; 75 min after inoculation, the bacterial CFU were below detection limit) meaning that the escaped bacteria would not be able to divide and dilute the reporter signal. We have also confirmed this in *in vitro* experiment shifting bacteria from the MgMES pH 5.0 medium mimicking the conditions in the SCV to DMEM + 10 % FCS + 20 µg/mL gentamicin (please see these results in response to the Reviewer’s 1 comment #12). However, we are grateful for the suggestion of using the TIMER-Bac system. We are currently integrating this system into our reporter cassette to study the escaped bacteria in more detail.

**Minor Issues:**

1) *Line 396: “we and others have observed a higher percentage of SPI-2 OFF bacteria following infection of both phagocytic as well as non-phagocytic cells” -> should read SPI-2 ON bacteria*

**Response:** Our claim comes from a similar comparison made by Rueter *et al*. (2021, Fig 1A vs B). However, we assume the confusion is coming from the unclear comparison and we have made this comparison clearer in the manuscript on current lines 421 to 422 (manuscript with highlighted changes).

2) *Figure 1H and 1I: What is the explanation for 20% more SPI2-ON bacteria in epithelial cells compared to macrophages? A better control would be a ΔssrAB mutant to show that the reporter plasmid is not expressed under any other influence.*

**Response:** In Fig. 1H (macrophages), there are ~75% of SPI-2^ON^ bacteria, while in the Fig. 5I (epithelial cells), there are ~60% of SPI-2^ON^ bacteria as measured using the stable GFP reporter. We assume this difference is caused by differences in host cell response to *Salmonella* and differences in the environment within SCVs. SCV composition differs to some extent between macrophages and epithelial cells (Reuter *et al*., 2020, DOI: 10.1074/mcp.RA119.001841). Also, the acidification of SCV differs between macrophages and epithelial cells (Rathman *et al*., 1996, DOI: ; Drecktrah *et al*., DOI: 10.1111/j.1600-0854.2006.00529.x). This difference is not visible in the destabilized GFP-ASV reporter though, suggesting that it is caused early after bacteria entry into the host cells.

We added the Δ*ssrAB* mutant to fig F1H and F1I.

3) *All figures: Where appropriate, individual data points should be shown to facilitate interpretation of the biological variation.*

**Response:** We added individual data points to most of our graphs.

In view of the results of new experiments, changes to figures and other explanations described above and in the manuscript, we hope that you will find the revised version acceptable for publication in PLOS Pathogens.

We look forward to hearing from you in due course.

With best regards,

Ondrej Cerny
