# Supplementary material for: Bimodal expression of Type 3 Secretion System 2 enables cooperative virulence among intracellular Salmonella Typhimurium
Source: PLoS Pathog. 2025 Dec 1;21(12):e1013728. doi: 10.1371/journal.ppat.1013728 (PMC12677785; doi:10.1371/journal.ppat.1013728)
Supplement: Attachment — Submitted filename: Kambova et al_rebuttal letter 2.docx [file ppat.1013728.s016.docx]

**Editorial office PLOS Pathogens**

**Bimodal Expression of Type 3 Secretion System 2 Enables Cooperative Virulence among Intracellular *Salmonella* Typhimurium (PPATHOGENS-D-25-01347)**

31^st^ October 2025

Dear Editor,

Many thanks for the new Reviewers’ comments on the above paper and for giving us the opportunity to respond to them. Below we provide our responses:

**Reviewer #2:**

The revised manuscript has been improved considerably, and the authors have addressed most of the previous concerns in a thorough manner. I have a few minor issues that still require attention:

*1. The first author’s name has been corrected in the main author list; however, it still appears as Kambova, M. in Line 908, Line 944, Line 971, and Line 996. Please update these instances accordingly.*

**Response:** All four mentioned appearances of the name Kambova are linked to the BioRender licenses. Milada Pospíšilová (formerly Kambova) got married in late September 2025 before the resubmission of the manuscript, but after the licensing of the Figures 3, 5, 6, and 7 by BioRender. The licenses therefore still carry her maiden name and cannot be changed unless the schematics are modified, despite her BioRender profile already shows her married name.

*2. Regarding the previous comment: “Please clarify why an OD 1.8 is used to represent the late exponential phase, while a time point of 16 hours is used to represent the late stationary phase,” it would be clearer to use a consistent metric for different growth phases. Either represent all phases by OD values or by time points (hours).*

**Response:** We agree it would be better to use a consistent metric for different growth phases. However, this is not always possible due to technical reasons. Since the bacterial cultures grow slightly differently in individual LB batches, they reach OD 1.8 at slightly different times and defining late exponential phase by time would, therefore, not be precise. In contrast, at the late stationary phase, different LB batches support bacterial growth to different OD (ranging from ~ 4 to 5 in our experience) and we cannot therefore use OD to define the late stationary phase across long term in our laboratory conditions.

*3. In Line 269, please verify the spelling of “cellulo.” Ensure that it is correct in the context of the manuscript.*

**Response:** We have checked the spelling and usage of “*in cellulo*” throughout the manuscript and find it correct in the context of the manuscript. Since we did our experiments in individual bacteria in test tubes, in infection conditions using cell cultures, and in infection conditions using laboratory mice, we feel that it is important to distinguish between *in vitro* (in the test tube), *in cellulo* (infection of cell cultures), and *in vivo* (infection of laboratory mice).

In view of the explanations described above, we hope that you will find the revised version acceptable for publication in PLOS Pathogens.

We look forward to hearing from you in due course.

With best regards,

Ondrej Cerny
